# Operationalization of the physical frailty & sarcopenia syndrome: rationale and clinical implementation

**Published:** 2016-01-31

**Authors:** E. Marzetti, R. Calvani, M. Cesari, M. Tosato, A. Cherubini, M. Di Bari, M. Pahor, G. Savera, A. Collamati, E. D’Angelo, R. Bernabei, F. Landi

**Affiliations:** 1 Department of Geriatrics, Neurosciences and Orthopedics, Division of Orthogeriatrics, Teaching Hospital “Agostino Gemelli”, Rome, Italy; 2 Department of Geriatrics, Neurosciences and Orthopedics, Catholic University of the Sacred Heart School of Medicine, Teaching Hospital “Agostino Gemelli”, Rome, Italy; 3 Gérontopôle, Centre Hospitalier Universitaire de Toulouse, Toulouse, France; 4 Inserm UMR 1027, Université de Toulouse III Paul Sabatier, Toulouse France; 5 Geriatric Hospital, Italian National Research Centres on Aging, Ancona, Italy; 6 Department of Experimental and Clinical Medicine, Research Unit of Medicine of Aging, University of Florence and Azienda Ospedaliero-Universitaria Careggi, Florence, Italy; 7 Department of Aging and Geriatric Research, University of Florida – Institute on Aging, Gainesville, FL, USA

**Keywords:** aging, physical performance, functional impairment, skeletal muscle, disability, organ failure

## Abstract

Over the years, different operational definitions have been elaborated to identify frail older persons, but none of them has received unanimous consensus. This, in turn, has hampered the clinical implementation of frailty as well as the design of targeted interventions. To overcome the current limitations in the field, a novel operationalization of physical frailty (PF) is proposed which grounds its roots in the recognition of sarcopenia as its central biological substrate. This conceptualization is based on the fact that the clinical picture of PF overlaps substantially with that of sarcopenia. The two conditions may therefore be merged into a new clinical entity, the PF & sarcopenia (PF&S) syndrome, in which muscle loss represents both the biological substrate for the development of PF and a major pathway whereby the negative health outcomes of PF occur. All of the components defining the PF&S syndrome are measurable in an objective manner, which will facilitate its incorporation into standard practice. The recognition of a precise biological substratum for PF&S (i.e., skeletal muscle decline) also opens new venues for the development of preventive and therapeutic interventions.

## I. INTRODUCTION

Healthcare systems are increasingly faced with a growing population of older adults characterized by the co-existence of multiple, chronic disabling conditions [1]. The gap between the demand of effective intervention strategies and the availability of medical programs specifically dedicated to older adults results in inappropriate use of resources and escalating healthcare expenditures [1,2]. Functional dependence, in particular, poses a serious threat on the sustainability of healthcare systems [3]. Hence, although prolongation of life remains a major public health goal, specific strategies should be developed to preserve physical function into late life and maximize disability-free life expectancy.

In this scenario, the geriatric syndromes of frailty and sarcopenia have gained special interest due to their association with a number of potentially preventable adverse health outcomes. These syndromes may therefore well serve as paradigmatic conditions around which healthcare systems can be re-shaped and optimized to address the specific medical needs of older persons [4,5].

Despite the efforts of many researchers, frailty and sarcopenia are still “orphan” of firmly established operational definitions and evaluation methodologies, and this has so far impeded their translation into every-day clinical practice [6]. The ultimate consequence of these persisting uncertainties and debate is the lack of effective interventions to prevent the development and impede the progression of the two conditions. Such an *impasse* might be overcome through the development and validation of a conceptual framework specifically elaborated to facilitate the translation of frailty and sarcopenia in clinical practice. To this aim, the pathophysiological and clinical foundations of the two conditions should be precisely defined, in order to assist in the design and implementation of *ad hoc* preventive and therapeutic interventions. Such an ambitious task represents a major objective of the «Sarcopenia and Physical fRailty IN older people: multi-componenT Treatment strategies» (SPRINTT) study, a research project recently funded by the Innovative Medicine Initiatives (IMI) [7].

## II. PHYSICAL FRAILTY AND SARCOPENIA: AN INNOVATIVE THEORETICAL APPROACH

In the literature, different criteria have been proposed to identify frail older persons, which mainly refer to two conceptual models: the cumulative deficit approach, proposed by Rockwood et al. [8], and the frailty phenotype, proposed by Fried et al. [9]. Both models have received empirical validation; yet, the frailty phenotype has some recognizable advantages, which are probably responsible for its more frequent use in the geriatric literature. First, it has a solid, well-characterized pathophysiological background [9,10]; second, its definition, based on only five items, makes it more easily applicable than the complex and cumbersome cumulative deficit approach; third, and most importantly, it identifies frailty at an early stage, before overt disability has developed, therefore opening venues to preventing interventions. The condition depicted by the frailty phenotype is, indeed, predictive of major negative health-related outcomes, including disability in the domains of mobility and self-care (basic activities of daily living), institutionalization, and mortality [10].

Although the pathways leading to the development of frailty and its progression towards disability are complex, and encompass a variety of mechanisms besides those centered on muscle structure and function, the frailty phenotype shows substantial overlaps with the clinical picture of sarcopenia [11]. Indeed, physical function impairment represents the inner core shared by the two conditions. Based on this observation, a novel operationalization of physical frailty (PF) is proposed, which recognizes sarcopenia as its central biological substrate [8]. According to this proposition, the decline in muscle mass and function during aging would represent the pathway through which the negative health outcomes of PF ensue [12]. To put it differently, sarcopenia may be envisioned as the “organ failure” underlying the clinical manifestations of PF [11]. It follows that the two conditions may be merged into a new clinical entity, the PF & sarcopenia (PF&S) syndrome, with important implications for its clinical implementation and the development of dedicated healthcare initiatives.

Notably, all of the components describing the PF&S model are measurable and quantifiable. The implementation of such a paradigm would therefore allow the accurate operationalization of PF&S, a clear identification of the affected population, and the rapid translation of research findings to the clinical arena. Such a conceptualization would also make PF&S comparable to other chronic degenerative conditions of old age (e.g., chronic obstructive pulmonary disease and congestive heart failure) because mirroring the paradigm of a biological substratum for a specific set of symptoms/signs determining a measurable decreased function [11]. The PF&S syndrome may thus gain its spotlight among the geriatric “giants” [13], besides becoming easily acceptable by healthcare professionals, public health authorities, and regulatory bodies.

## III. THE PF&S SYNDROME: FROM “BRAINSIDE” TO BEDSIDE

**Fig. 1.**
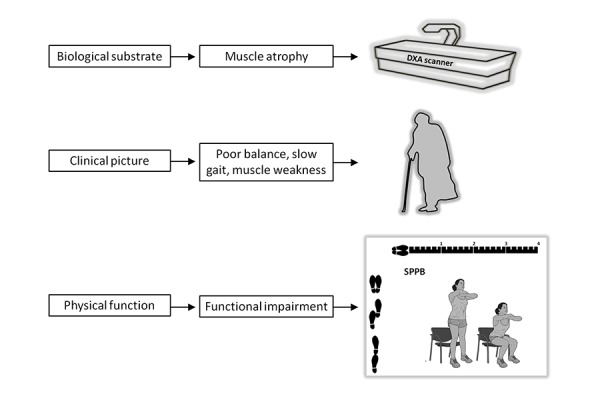
**Physical frailty and sarcopenia (PF&S): from concept to practice.** The PF&S condition can be objectively measured in all its domains, spanning from biologic substrate (reduction in muscle mass) up to its clinical manifestations. The muscle domain can be measured using standard techniques, such as dual energy X-ray absorptiometry (DXA). The clinical “identikit” of an older person with PF&S can be sketched as the “perfect storm” of poor balance, slow gait and muscle weakness. This picture can easily be quantified using specific tools, such as the Short Physical Performance Battery (SPPB).

As previously elaborated, the proposed conceptualization of PF&S involves a set of objectively measurable domains that encompass the full spectrum of the condition, from biological substrate to clinical framing (Figure 1). The muscle atrophy component can easily be quantified with available techniques (e.g., dual energy X-ray absorptiometry [DXA]). Through the adoption of Classification and Regression Tree (CaRT) models, the Foundation for the National Institute of Health (FNIH) initiative has recently identified cut-points of low lean mass that are associated with increased risk of mobility disability [14,15]. It appears then reasonable that FNIH criteria, rather than consensus definitions of sarcopenia, be used to identify the muscle atrophy component of PF&S.

At the clinical level, the manifestations of PF&S (e.g., impaired balance, slow gait, muscle weakness) can be measured in an objective manner using specific tools, such as the Short Physical Performance Battery (SPPB) [16–18]. This tool has shown to predict major negative health-related events (including disability, institutionalization, and mortality), and also provides an accurate picture of the “biological age” of an older person [16–19]. At the same time, the SPPB is strongly related with the quantity and quality of skeletal muscle, and is therefore able to capture the core of PF&S [4]. In particular, a cutoff of 9 (included) at the SPPB allows the identification of older adults at increased risk of adverse health events, including reduced quality of life, disability and mortality [16,17,20].

## IV. CONCLUSION

The ongoing demographic transition is accompanied by substantial changes in medical needs and nosographic scenarios, which imposes the development of counteractions against highly prevalent disabling conditions. The traditional paradigm of a “standalone-disease medicine” has clearly become outdated in a clinical world dominated by older persons suffering from multiple chronic conditions and mutually interacting syndromes [21]. On the other hand, although the transition from the disease-centered model of care to a “holistic” approach is crucial in geriatric care, the identification of pathophysiological mechanisms altering key functions for the older person (e.g., physical function) and potentially usable as targets for interventions are still necessary. The operationalization of PF&S herewith proposed surpasses the traditional paradigm of healing through treating a single disease, and may support the design of future nonpharmacological and pharmacological strategies aimed at improving the older person’s health status by focusing on the functional domain [11,22,23].
